# Machine learning modeling practices to support the principles of AI and ethics in nutrition research

**DOI:** 10.1038/s41387-022-00226-y

**Published:** 2022-12-02

**Authors:** Diana M. Thomas, Samantha Kleinberg, Andrew W. Brown, Mason Crow, Nathaniel D. Bastian, Nicholas Reisweber, Robert Lasater, Thomas Kendall, Patrick Shafto, Raymond Blaine, Sarah Smith, Daniel Ruiz, Christopher Morrell, Nicholas Clark

**Affiliations:** 1 Department of Mathematical Sciences, United States Military Academy, West Point, NY 10996 USA; 2 Department of Computer Science, Stevens Institute of Technology, Hoboken, NJ 07030 USA; 3 Department of Biostatistics, University of Arkansas for Medical Sciences, Little Rock, AR 72205 USA; 4 Arkansas Children’s Research Institute, Little Rock, AR 72202 USA; 5 Army Cyber Institute, United States Military Academy, West Point, NY 10996 USA; 6 Department of Mathematics and Computer Science, Rutgers University, Newark, NJ 07102 USA; 7 Department of Electrical Engineering and Computer Science, United States Military Academy, West Point, NY 10996 USA

**Keywords:** Risk factors, Metabolism

## Abstract

**Background:**

Nutrition research is relying more on artificial intelligence and machine learning models to understand, diagnose, predict, and explain data. While artificial intelligence and machine learning models provide powerful modeling tools, failure to use careful and well-thought-out modeling processes can lead to misleading conclusions and concerns surrounding ethics and bias.

**Methods:**

Based on our experience as reviewers and journal editors in nutrition and obesity, we identified the most frequently omitted best practices from statistical modeling and how these same practices extend to machine learning models. We next addressed areas required for implementation of machine learning that are not included in commercial software packages.

**Results:**

Here, we provide a tutorial on best artificial intelligence and machine learning modeling practices that can reduce potential ethical problems with a checklist and guiding principles to aid nutrition researchers in developing, evaluating, and implementing artificial intelligence and machine learning models in nutrition research.

**Conclusion:**

The quality of AI/ML modeling in nutrition research requires iterative and tailored processes to mitigate against potential ethical problems or to predict conclusions that are free of bias.

## Introduction

Complex, large, and multimodal nutrition datasets are being aggregated for the purpose of advancing personalized nutrition, such as the Personalized Responses to Dietary Composition Trial-1 (PREDICT) study [1], a study focused on nutritional prediction of glycemic responses [2], and the new Nutrition for Precision Health program [3]. Such studies and programs highlight a critical need and growing desire to implement machine learning (ML) in nutrition research. For nutrition researchers new to ML but well-versed in statistical methods, using ML models will require adhering to best practices from statistical methods while establishing new approaches that address the complexities of ML models.

The availability of AI/ML capabilities in commercial software packages has made AI/ML algorithms accessible to the wider nutrition research community. However, the high accessibility of AI/ML models through “click and play programs” belies their complexity, which, when overlooked, can lead to myriad unanticipated ethical problems that violate published AI principles [4, 5]. Standardized procedures for the appropriate implementation of ML models often do not exist. Deceptively simple questions, such as whether the sample size is adequate for model fitting, often require iterative evaluation by the modeler that cannot be built into standardized software. Failure to follow a reflective thoughtful approach to AI/ML modeling can lead to errors and biased conclusions that can have deleterious results [6].

Herein we define ML as computer algorithms that improve automatically through experience [7, 8]. The closely related term “artificial intelligence” (AI) is often interchanged with ML. AI refers to an algorithm that can learn insights, adapt through feedback, be dynamic, respond to its environment, and problem solve independently with minimal human supervision [8, 9]. ML is sometimes considered a subset of AI and vice versa, and the terms are frequently used interchangeably [8]. We, therefore, refer to both types of algorithms as AI/ML because many of the ethical concerns discussed herein apply regardless of distinction.

The Alignment Problem by Brian Christian [6] and landmark studies like those of Buolamwini and Gebru [10] highlight many unfortunate consequences of launching ML models without careful examination of the data used for modeling, without application of more than one modeling approach, and without a thorough review and surveillance of model predictions and conclusions. Such negative consequences can range from racial or other discriminatory predictions, wasted time or opportunity, negative health outcomes, or even death. Many detrimental consequences of AI/ML applications covered in Christian’s book can be summarized as resulting from poor modeling practices. In addition, a recent review of 62 studies that used machine learning to detect and predict COVID-19 from chest radiographs and CT scans found that every single study had a methodological flaw [11]. These flaws ranged from lack of transparency regarding how key modeling decisions were made to not including model validation experiments [11].

With many and varied approaches available for evaluating AI/ML models, how can nutrition modelers, manuscript reviewers, and journal editors ensure that the models are complete, minimize predictions or conclusions that can cause patient harm, avoid bias, and minimize ethical violations [12]? While we cannot address every possible situation and scenario that could arise, we address common considerations that nutrition researchers may encounter when developing and/or evaluating AI/ML models. The considerations we address herein came from our experience as AI/ML modelers in nutrition, serving as reviewers of AI/ML modeling articles, and our service as editors for top nutrition research journals. We frame the discussion for an audience of nutrition researchers who are familiar with statistical and ML methods in nutrition research but may be new to or have limited experience with developing, evaluating, or implementing AI/ML models.

The description and recommendations here build upon an existing body of literature. The Findable, Accessible, Interoperable, and Reusable (FAIR) Data Principles [13] involve stewardship and management of data which have some overlap with AI/ML best modeling practices. There have been several articles on best AI/ML modeling practices which draw upon and integrate with FAIR principles [14, 15]. Articles that provide overviews of machine learning also include some best modeling practices [16, 17] and articles that are specific to an application like image analysis [18] include best modeling practices that scale to other disciplines. In addition, discipline-specific checklists are now being applied for publications such as the Checklist for Artificial Intelligence in Medical Imaging (CLAIM) [19], the machine learning checklist for Neural Information Processing Systems [20], and the machine learning reproducibility checklist produced by the Computer Vision and Pattern Recognition Conference [21]. The guidelines and checklist presented here focus on the viewpoint of a nutrition researcher who has a background in statistics and wishes to build on that background to include AI/ML models to describe, predict and explain nutrition data.

We begin with some well-known modeling practices derived from statistical methods that extend to AI/ML modeling. We next move to two important areas specific to AI/ML model development: appropriate sample sizes and balanced datasets. Next, we address the need for simultaneous development of models and specifically explainable AI/ML models. Finally, we emphasize the need for increased data literacy. With the application of new and complex AI/ML approaches in nutrition research, we as a community need to learn more about the underlying properties, requirements, capabilities, and limitations of AI/ML model development. Because AI/ML approaches are relatively new [1, 2] in nutrition, many of the examples of bias and error arising from poor development and evaluation of AI/ML models are drawn from other disciplines. These examples, while not specifically in nutrition, provide can raise our awareness of potential pitfalls as a higher dependence on AI/ML models in nutrition research advances. Table 1 serves as a Table of Contents, and Table 2 is a checklist that summarizes our tutorial. The checklist in Table 2 is presented in order of AI/ML execution starting with study design and ending with model evaluation. While every step in the checklist is important as a best practice, the most important result of the checklist is reproducibility. If we consider the AI/ML modeling process analogous to the methods behind the experiment, the checklist provides clear, rigorous, and transparent guidelines for the methods that ensure the results are reproducible.

**Table 1:** The Table of Contents is hyperlinked to ease navigation to sections within the article.

Hyperlinked Article Sections
Introduction to Extensions of Statistical Methods to AI/ML
Measurement Error
Selection Bias
Sample Size Calculations for AI/ML
Missing Data in AI/ML
Data Imbalance
Explainable AI
Data Literacy: The AI User Responsibility

**Table 2:** Checklist for ethical and effective application of AI/ML modeling in nutrition research.

Considerations for Ethical & Effective Application of Machine Learning for Nutrition Research		
Item No.	Item	Recommendations
Study Design		
1	Describe Overall Goal	Is the purpose to understand new data, select the most informative features from data, inform other researchers/clinicians/public, develop a model that makes predictions or diagnoses, or something else?
2	Describe Data	Clearly identify
		1. The data source
		2. When data were collected
		3. Over what time period data were collected
		4. Whether data will continue to be collected
		5. The size of the dataset
		6. The collection methodology
		Disclose in the manuscript if any of the above are unknown.
		Describe
		1. The approaches for minimizing measurement error during data collection
		2. The approaches for minimizing collection procedure bias
		3. Warning labels regarding the representation of the data
		If existing data sources are used, why were these particular source(s) used?
		Do the data represent the target population(s) (the population that your AI/ML models will be used to predict) accurately?
4	Discuss AI/ML Suitability	Are the modeling approach(es) supervised or unsupervised? Will the models be updated with additional data and, if so, how?
		Explain the suitability of AI/ML to answering the question. For example, is there an abundance of complex data? What were the results of traditional approaches such as regression? Do you suspect the data contain underlying patterns or correlations that a computer could learn?
5	Establish Evaluation Criteria	What evaluation criteria will you use to assess the performance of your model(s)?
		Why did you settle on these criteria? If using categorical classification, report a confusion matrix in the results. In the discussion, explain what is the impact is of the false positive rate and false negative rate on your application. If you are predicting continuous outcomes, what is the cost of over or under-estimating?
Data Pre-Processing		
6	Handle Missing Data	What data are missing? What techniques were employed to account for missing data? If multiple techniques were used, how were they evaluated against each other?
		In the discussion, describe the potential cause for missing data. Are the data MCAR (Missing Completely At Random), MAR (Missing At Random), MNAR (Missing Not At Random)?
		Why did you settle on a particular method for handling missing data?
7	Classify Outliers	How were outliers defined? Were outliers removed? What is the impact of including the outliers on your modeling? Why did you settle on this method to identify and classify outliers? Simulate the potential comparison of model performance with/without outliers or outliers defined by different approaches.
8	Balance Classes	Did you balance the subgroups used as inputs or the classes you are predicting? What was the method used to balance the dataset (e.g., up-sampling previously untapped populations)? Justify the choices of balancing classes. For example, did the initial class distribution fail to match the distribution of the population for which you are applying the AI/ML model? Describe your balancing methodology, including justifying why not balancing would be appropriate if you choose not to balance your dataset.
9	Select Features	Which features from your dataset did you select for AI/ML model training? Were all available features used or a subset? Explain why the features were selected.
10	Evaluate Dataset Size & Augmentation	Was the dataset reduced/expanded through resampling or augmentation?
		Is the dataset of an appropriate size for the AI/ML modeling methods? Why was the dataset reduced or expanded? Are you targeting a particular AI/ML algorithm (i.e., neural network)?
		How will the size of the dataset, after pre-processing, inform the choice AI/ML algorithm(s) (see next section)?
Algorithm Construction		
11	Select Algorithm(s)	List all AI/ML modeling approaches that were trained and evaluated.
		Justify why the approaches were selected. If only one approach is used, explain why it was not feasible or not desirable to test more than one model. Is at least one AI/ML approach explainable? If not, why?
		Clearly assess assumptions of the AI/ML models and describe whether they hold.
12	Algorithm Explainability	Describe approaches to enhance or select for explainability of models.
		Describe the level of explainability of the selected models. Can the model’s decision-making be understood or interpreted?
		If selecting a non-explainable model, justify the choice (e.g., far superior model performance when non-explainable). If an explainable model was not paired with the non-explainable example, provide justification.
13	Model Reproducibility	Provide the exact hardware, software, and hyperparameter specifications used to train AI/ML model(s). Supply the algorithms, data, and code to reproduce the model. Explain the steps required to reproduce the results. If appropriate, explain why data, code, software, or other artifacts necessary to reproduce the work are not publicly available.
Algorithm Evaluation		
14	Determine Baseline Performance	Determine Comparison Performance. What is the state of the model accuracy in the literature? How are you improving understanding or accuracy beyond what already exists?
15	Internal Validation	Describe how the training and validation/test set were divided and why. Use evaluation approaches like k-fold cross-validation to capture internal variance. Include multiple runs on any machine learning model that relies on random initiation of weights or other model parameters (e.g. neural networks).
		Do the results suggest the model was overfitting or underfit? Did you use internal validation approaches like k-fold cross-validation? If so, what were the results?
16	Determine Best Model	Identify and explain which AI/ML model performed the best in accordance with your chosen evaluation criteria.
17	External Validation	Describe any approaches used to externally validate the model (i.e., model validated on an independent sample).
		If not externally validating, why not? If externally validating, explain why that external data source and approach are being used.
		What are the results of external validation? Alternatively, what are the ramifications of not externally validating?
18	Model Deployment Considerations	Describe how the model will be deployed and who the end user would be.
		Describe the use cases for the model. What are the limitations of the model? How often should it be reevaluated or retrained? What is the shelf life of the data?
19	Considerations	Offer considerations for future research. What additional techniques or data could be tested?

## Extensions to AI/ML from statistical modeling

Statistical modeling has well-developed methods for identifying, mitigating, and transparently reporting bias and error. We distinguish “bias” in the statistical sense from “bias” in the social sense. When we discuss bias in a model, we are indicating that the expectation of the model does not match the true value; that is, we reliably come to inaccurate conclusions. More specifically, we are referring to bias that comes from the statistic being used to estimate a parameter, or we are discussing bias that arises from using data that is not representative of our population. In either case, the result of the bias is a parameter estimate that is not accurate. However, we should note that all bias is not bad; statisticians will often use a biased estimator if it results in a lower mean squared error such as what is used in the popular LASSO algorithm. Biased data, or sampling data that is not reflective of our population, on the other hand, is rarely a good idea and can lead to disastrous results if not properly accounted for. This is different than the social aspects of bias, such as prejudice. Unfortunately, some forms of bias discussed herein (attrition bias, selection bias) may result in or result from socially biased research approaches, which in turn can create a model that inherits those biases, and ultimately creates a statistically biased model. Many of the statistically-based quality assurance checks still apply and are even more important to consider when developing machine learning models. Unfortunately, these statistical best practices are oft “forgotten” [22] and are not standard or routine when reporting the results of machine learning predictive models. Identifying whether the characteristics of participants who dropped out were different than completers, whether missing data were missing at random, or expressing limitations on extending predictions beyond the sample are common omissions [23, 24].

Statistical modeling best practices that ensure data are collected in manners that reduce bias and errors exist and are also relevant for AI/ML model development. It is not our intent to provide a comprehensive statistical tutorial on the statistical methods. Instead, we provide a summary of bias and error that is often observed in nutrition research and address how statistical mitigation strategies also prevail for AI/ML models. Some methods are “best (but oft-forgotten) practices” [25] and we recommend the statistical series at the *American Journal of Clinical Nutrition* for an in-depth tutorial into statistical practices frequently applied in nutrition research [22].

### Measurement error

#### Take home message

Controlled data with minimal measurement error are needed as a gold standard to compare clinically relevant data that the models will be used on. Explainable AI/ML models are key to understanding the propagation of measurement error.

##### What is it?

There is a wide range of measurements in clinical nutrition. Measurements of glycated hemoglobin (HbA1c) are objective and correlate to a patient’s diabetes status [26]. On the other hand, measurements that are obtained from accelerometers are also objective, but can be extremely noisy and are not able to estimate physical activity expenditure well in comparison to gold standard methods [27]. However, the largest source of measurement in nutrition research, self-reported energy intake, is not objective and sometimes not reliable without triangulating with other methods [28] for deriving scientific conclusions [29–31]. There are numerous additional diverse measurements in nutrition research such as clinical energy balance measurements [32, 33], body composition [34], anthropometry [35], and biomarkers [36]. Within these measurements, some of the measurement errors occur at random while some are systematic or idiosyncratic.

Statistical modeling has long included discussions of error, including assumptions about the nature of the error (e.g., normally distributed with zero mean) that have to be satisfied in order to make statistical inferences and methods that assume that the true values are measured with error (e.g. Bayesian error models) [37, 38]. Because measurement error can render the results of a study or model meaningless, imprecise, or unreliable [39], there is a vast literature on handling measurement error [40, 41] in the context of statistical modeling.

##### What should we do about it?

While we cannot eliminate all measurement errors, there are best practices to reduce measurement errors during data collection. Some best practices to minimize measurement error is to take multiple measurements of the same variable when possible and to collect the data with precision. For example, body weights should be collected under similar conditions, such as first thing in the morning, on the same scale, and in a hospital gown. To obtain information on the variation in measurements, the measurement should be taken multiple times (e.g., three times for body weight). How much measurement error is in the input data needs to be conveyed, not just in peer-reviewed publications, but also as “warning labels” in data repositories that will include AI/ML prediction tools. An exemplar for including warning labels within a data repository is the All of Us Research Program [42], which alerts data users to the quality and distribution of the data during access. A robust list of resources for tagging data for reuse and reproducibility appears on the Go FAIR website for the Findable, Accessible, Interoperable, and Reusable (FAIR) principles [13, 43].

In the case of non-objective measurement error, it has been suggested that self-reported dietary intake should not be used as true dietary intake to derive scientific conclusions [29, 30]. This does not mean that self-reported dietary intake data is not valuable during interventions. There are examples of self-reported dietary intake data being used in tandem with other tools such as energy intake wearables [44, 45] and mathematical models that predict weight loss to guide intake [46] improving dietary adherence even more than any of the dietary assessment methods used alone [28]. The danger of using data like self-reported dietary intake as true intake to train AI/ML models is that the models will identify patterns that are artifacts of error from the input data which will then be used to make erroneous predictions that inform decision-making. For example, intake has been found to be underreported in individuals with obesity [31, 47], which has led to erroneous predictions and conclusions that people with obesity gain weight while eating less [48]. It is important to note that if we knew the bias in the self-reported data this could easily be corrected. Future research should focus on identifying the magnitude and direction of biases in the data using proxy or alternate datasets. Multilevel models also serve as potential tools that should further be studied to determine how they can potentially be leveraged to correct self-reporting biases [49].

We also need to be concerned about the measurement and its error under conditions of research versus conditions of use. Using body weight as an example: if a model is trained on body weight collected under exacting conditions, multiple times, at the same time of day, the model may not perform as well when using body weights taken at the clinic once, at any time of day, often without removing excess clothing. The measurement for the model thus does not match the measurement for use.

##### Extension to AI/ML modeling

Errors in measurement have the potential to result in erroneous decisions. Simple models allow us to track how error propagates from the initial variable to the final output. In comparison to simpler explainable models like linear regression, it is often challenging to track error propagation in AI/ML models when they contain nonlinearities and interconnections between variables that are not immediately apparent, also known as “black boxes” [50]. Furthermore, AI/ML methods often incorporate nonlinear aspects which tend to exacerbate error [51]. Specific methods to address individual AI/ML models exist, but there does not exist a one size fits all solution to generally characterize error propagation within AI/ML models [51]. The reliability of a model where the error propagation is unknown cannot be properly characterized; however, model developers can look to the literature for the specific model to find methods to quantify error propagation [52].

### Selection bias

#### Take home message

Characteristics of the dataset, such as demographics, need to be summarized and explored for limitations prior to training algorithms. Justification should be provided for why the AI/ML model is appropriate for the sample size. Approaches such as up-sampling and down-sampling can be cautiously applied using an iterative process to mitigate concerns about selection bias.

##### What is it?

One of the most well-known examples of selection bias in artificial intelligence occurred when a Google Photos image classifier incorrectly identified people of color as gorillas [6]. Google attempted to fix the artificial intelligence model from a top-down approach relying on various strategies; however, the underlying problem was that the model training dataset did not contain enough people of color. This is known as “selection bias”. Selection bias occurs when the individuals or groups in a dataset differ from the population of interest in a systematic way [53]. In the Google Photos example, the data on which the model was trained did not fully represent the population the models were applied for. As summarized by Brian Christian, the problem with “a system that can, in theory, learn just about anything from a set of examples is that it finds itself, then, at the mercy of the examples from which it is taught” [6].

##### What should we do about it?

Selection bias awareness is required in both study design and in reporting model capabilities. When recruiting, investigators should focus on the population they hope to generalize to and then recruit participants that meet those criteria. Recruiting a population that aligns with the target population for study outcomes will minimize selection bias. However, such recruitment may require creative ways to reach previously untapped populations [6].

##### Extension to AI/ML modeling

Recruiting representative populations for training datasets may not always be possible. For instance, large datasets may consist of convenience samples like electronic health records [54]. One method to account for this limitation is to weigh the data for key characteristics between the sample and population of interest. Weighting the data for regression applications is straightforward, but does not extend to AI/ML models that are often nonlinear. An extension of the statistical weighting approach to AI/ML models is to “up-sample” or “down-sample” the data according to weights. For example, if the dataset contains a sample of 20% females and 80% males, “up-sample” by repeating the 20% observations until the dataset female:male ratio matches the population of interest (e.g., ~50%). Conversely, a random sample of male subjects can be selected to down-sample or develop a dataset that contains the target female:male ratio. While this concrete example addresses female:male imbalance, it does not address other potential imbalances. For example, the female sample may have a BMI distribution different from the population (e.g., the sample is all below 25 kg/m^2^). AI/ML models may therefore incorrectly learn that females will have BMI below 25 kg/m^2^ without appropriately addressing imbalance. In all cases, the limitations of the data used to train the model should be made explicit in publications and any software application or tools used to disseminate the model should warn the user of limitations such as the characteristics of the training dataset.

## Considerations specific to AI/ML modeling

### Sample sizes calculations

#### Take home message

No one-size-fits-all approach exists to calculate sample sizes for AI/ML models. Adequate sample size depends on the application and model complexity. Sample size calculations for specific AI/ML models often require an iterative process. For reproducibility, the justification for the sample size always should be articulated.

##### What is it?

Having a large enough sample to train and test AI/ML models is critical to avoid overfitting or underfitting models. The definition of model overfitting is when the model fits too closely to the training dataset [55], thereby capturing idiosyncrasies of the observed data rather than generalizing true data properties. Ethical issues with overfitting occur when models perform well on the training dataset, but do not translate well to new data. For example, an overfit model that uses biomarkers to predict patient health will predict accurately the patient’s health used in the sample to develop the model, but misdiagnose patients not used in model development as being healthy when they actually require treatment [56]. There are several ways to mitigate potential overfitting and sample size can play a role. In general, the more complex the model (e.g., more weights, input variables, and layers in a neural network), the more data required to avoid situations like overfitting. Underfitting, on the other hand, can occur when there is not enough complexity in the model to match the supplied data [57]. In both cases, selecting the right sample size depends on the complexity of the model, tests for goodness of fit in independent data, and iterative evaluation of the model design versus model’s outcomes. In addition, in AI/ML models that are used for feature selection or identifying which variables are relevant, too small of a training dataset may result in lower data variability and, consequently, degrade the identification of important features [58].

##### What should we do about it?

Power is the probability of detecting a difference when one really exists (that is, one minus the probability of making a type 2 error). In statistical analyses, it is used to determine the sample size required to make appropriate and corresponding statistical inferences. Although well-studied in the area of AI/ML modeling [59], a similar systematic and tractable method to determine sample sizes for AI/ML models cannot be provided. The nonlinearity and complexities of AI/ML models and the multiple models that fall into the category of AI/ML do not lend well to a uniform process for calculating sample sizes when compared to more simplistic analyses like a t-test. Despite these challenges, several published “rules of thumb” exist [60]. For classification models (e.g., decision trees or neural network classifiers), a rule-of-thumb is that the sample size needs to be at least 50–1000 times the number of classes being predicted [61]. For example, if you are predicting categories of obesity (BMI ≥ 30 versus BMI < 30), this is a binary classifier and your sample size would need to be between 100 and 2000. Similar rules of thumb exist relating sample sizes to the number of input variables or features, or sample size to number of weights used in the model. These rules ultimately relate the sample size to the complexity of the model (e.g., number of classes predicted, number of variables used as inputs, the number of hidden layers, or number of weights) and range widely as demonstrated with the 100–2000 range for a binary classifier. Thus, an iterative process is required to determine the appropriate sample size tailored for each individual problem and model. In publications or other forms of model dissemination, the sample size choice must be justified and clearly articulated.

For exploratory modeling when the number of covariates is high compared to the number of data points, regularization techniques such as LASSO regression or, more generally, Elastic Net regression offer ways to fit data. Here the resulting parameters will be biased, however, more complex models can be fit [62]. Whether these techniques are appropriate depends on the overall goal of modeling, but they are often good tools if practitioners are attempting to both diagnose a root cause as well as build a predictive model.

### Missing data

#### Take home message

Nutrition research frequently includes missing data, such as from incomplete self-reported habits or missed clinical visits. How we handle missing data can influence AI/ML model predictions and conclusions. In addition to traditional statistical approaches for handling missing data such as imputation, methods using AI/ML models have been developed to handle missing data. In some cases, missingness can be treated as a model feature. Lack of adherence to prescribed interventions and other reasons for missingness can be captured using this approach.

##### What is it?

Missing data are pervasive in healthcare and especially common in nutrition research. Missing data can occur in multiple ways. Nutrition research often relies on logs kept by human subjects or surveys (such as the food frequency questionnaire (FFQ), food diaries, or 24-hour recalls) [63]. Individuals may forget to record a specific meal, selectively omit information due to desirability bias [64], or fail to complete the dietary instrument altogether. Objective measures, too, may have missing data, such as missed samples for biomarkers or user and technological errors failing to record behaviors. Datasets may therefore be missing individual data points (e.g., a meal), entire variables (e.g., no blood glucose data), or specific time windows (e.g., losing a day of data due to technology failures).

There are three main types of missing data and each has different implications for data analysis [65]. The first is missing completely at random (MCAR). An example of this is if a researcher is out sick and misses follow-up appointments with some subjects. The probability of a data point being missing is then independent of any characteristics of the participants. MCAR data reduces the sample size (and study power) depending on the proportion of missing information. In some cases, information for some missing data can be inferred from other information in the dataset. However, many models can use only complete records, but in the case of MCAR, ignoring missing data will not lead to biased results. This type of missingness is unlikely. A more common scenario is data that is missing at random (MAR), which is when the likelihood of a variable being missing depends on other variables [66]. For example, if someone leaves out snacks in their meal logs only on days when they do not exercise, data on snacks would be MAR. Similarly, if people are more likely to answer survey questions based on their age or gender, those data would also be MAR. If we use only complete records with MAR data, we may get a biased estimate of how prevalent something is in the population (e.g., 100% of people who snack exercise). For some types of analysis, such as likelihood-based methods, this type of missingness is considered ignorable, though this terminology is a misnomer. We cannot ignore that missingness depends on other observed variables and cannot use only complete records without introducing bias. For causal inference, using only complete records can mean we fail to discover causal relationships (e.g., without any variation in reported snack behavior we cannot find what causes it). Finally, when the presence of data depends on the variable of interest itself, data is missing not at random (MNAR). An example of this is if people only self-report their weight when it falls in certain ranges if doctors measure HbA1c when they suspect it is high, or if an individual with diabetes tests their blood glucose only when they suspect it is too high or too low. Ignoring incomplete records will lead to biased results. For example, ignoring times without glucose values will give the impression that glucose is always at an extreme. Predictive models trained on datasets with data that are MNAR will fail when used in the real world, since they will have few examples of glucose values outside of the extremes. Finally, note that statistical tests to distinguish whether missing data are MCAR, MAR, or MNAR are often highly limited.

##### What should we do about it?

Ignoring subjects who dropped out of a clinical trial can bias results [66], and the same is true for AI/ML methods. Failing to account for missing data can lead to incorrect results and models that fail when applied to new populations. The primary strategies for handling missing data are imputation or modeling the missingness. The majority of imputation methods are designed for data that is MAR, and use observed values to reconstruct missing ones. The simplest approach, using the mean (or mode) value in the observed data to replace missing values, has been used widely, but has significant limitations and is not recommended for use in nutrition studies. The mean recorded bodyweight or calorie intake in a dataset is simply not representative of missing instances. Similarly, carrying forward the last observation (e.g., assuming someone’s bodyweight is the same until it is next recorded) requires assumptions about the stability of these variables that are not justified. More advanced approaches, such as k-nearest neighbor (kNN), aim to find similar observed instances to missing ones, and have been applied to FFQ data [67]. Rather than using a population average, kNN finds the most similar subjects to one with missing data, and uses a function of their values to replace missing ones. Note that this approach is only appropriate for MAR data, where there is a relationship between observed values and missing ones. A limitation is that accuracy declines as more variables are missing for an instance, and it cannot be used when all data is missing (e.g., for time series data, if all variables are absent at one-time point). Multiple imputation [68] allows modeling of uncertainty in missing data. Rather than fill in gaps with a single value, these methods create multiple imputed datasets. Combining results on each enables estimates of error due to the missing data. This approach has been used on FFQ [69], 24-h recall [70], and food log data [71]. For data that are MNAR, fewer methods exist, though some have been introduced to model data with variables that may be MNAR or MAR [72].

Notably, missingness can be informative and has been used as a feature to improve prediction. Intuitively, if a doctor chooses not to run a test or a person decides not to record a specific meal, those events are likely to be different from the ones that are observed. Thus, if we impute values for missing data, but do not capture the fact that data was not originally recorded, we may lose valuable information. Lin and Huang [73] showed that including indicators representing missing data improved predictions from electronic health record data. This has been repeated using other methods such as recurrent neural networks [74, 75].

### Data imbalance

#### Take home message

Datasets used for training must be balanced so models learn what and how input features are important to the application of the AI/ML model. The definition of balance will depend on the model type and intended application, but should consider the distribution of classes in a dataset. There are methods to “balance” a dataset that should be applied cautiously. For reproducibility and transparency, the percentage of different classes available in the training data as well as steps taken to balance the data need to be articulated.

##### What is it?

Data imbalance occurs when most instances in a dataset belong to a single or small subset of the total classes. For example, if females represent only 20% of a training dataset and males are 80% of the dataset, then we would say the dataset is imbalanced. Similarly, if a specific outcome of interest occurs at lower rates than all other outcomes, such as pregnancies complicated by gestational diabetes, and we are developing an AI/ML model to predict which pregnancies result in gestational diabetes, the dataset is also referred to as imbalanced.

In the case where a sub-group is smaller in size than other groups, AI/ML models “see” the subgroup less when learning. The lack of exposure can result in poor performance when restricted to the subgroup. This is exactly what occurred in the Google Photo example described in the Selection Bias section. While people of color were contained in the large dataset, the learning models did not see enough examples of people’s faces to be able to recognize faces of people of color when presented with a new photo.

In the second case, where the outcome occurs less frequently, such as gestational diabetes mellitus (GDM), failure to balance the dataset could result in flawed or non-informative models. It is estimated that GDM prevalence is between 4 and 10% of all pregnancies in the United States [76]. An AI/ML model that classifies GDM pregnancies would need more than 90% accuracy to outperform the model that assumes that GDM does not occur. This is because in the worst-case estimate of 10% prevalence of GDM pregnancies, the model that assumes GDM never occurs is already 90% accurate.

##### What should we do about it?

In the section on Selection Bias, up-sampling and down-sampling were already discussed and represent the most frequently applied method to mitigate problems with data imbalance. However, sampling up or down should remain an alternative to the original collection of balanced data. As mentioned earlier, up-sampling can result in AI/ML models learning artifacts of up-sampled observations that are not true features. Similarly, down-sampling the other classifications or subgroups reduces the size of the dataset to the smallest-sized subgroup.

## Application of explainable models

### Goals of explainable AI

The challenge with modern AI/ML models is that oftentimes the complexity of the modeling approach comes at a cost of explainability. This becomes an issue when practitioners attempt to draw causal or suggest causal relationships between predictors and response variables in the model. Because there are many AI/ML modeling approaches, one of the most important best practices is to use more than one AI/ML method and specifically to combine non-explainable with explainable models. For example, neural network classifiers are sometimes referred to as “black boxes” because while neural networks may have high accuracy for prediction, their complexity results in loss of explainability. However, using neural networks in tandem with an explainable method like logistic regression can circumvent the black box and provide explainability.

In general, to understand what elements of a model should be explainable it is useful to think of the Generalized Linear Models (GLM) framework. In this commonly used methodology a practitioner specifies a linear predictor that captures covariates of interest, a link function that maps the linear predictor to function of parameters in the statistical model, and a distribution function that captures the unexplainable parts of the model. The covariates, in this case, are the explainable part of the model. The practitioner may never explain why the uncertainty in the data follow, say, a gamma distribution, but they can explain the meaning behind how the explanatory variables are related to the response. Uncertainty then can further be partitioned through the use of Generalized Linear Mixed Effects Models (GLMM) that allow additional model-based uncertainty to be specified, therefore partitioning the uncertainty into model-based and data-based uncertainty. An interpretable AI algorithm should seek to behave similarly, where some key aspects of the model can be captured as a meaningful part of the parameter. In the machine learning literature tools such as Gaussian Process Regression have recently been used to model more complex data patterns than can be done using GLMMs but in an interpretable manner.

### Explainable AI

#### What is it?

AI/ML models have improved prediction beyond what was previously possible; however, due to model complexity, AI/ML models often lose internal model interpretability [77]. This loss of interpretability can eventually lead to unexpected and problematic model conclusions [6]. For example, deep convolution neural networks were trained using images of skin lesions, and they classified malignant versus benign melanomas with a high degree of accuracy when compared to the diagnosis of board-certified dermatologists [78]. However, it was later found that images of lesions that included rulers were classified as malignant because the model “learned” that when a ruler was included in the image, the lesion was more likely to be malignant. This artifact was introduced because rulers were included in images when the clinician already thought the lesion was more likely to be malignant [79]. If this artifact was not detected (that is, if the model was not explained), the model would have a high false-negative rate for new images. Explainable AI was promoted to preserve the high level of desirable accuracy that is provided by complex AI/ML models while retaining interpretation.

Explainable AI (XAI) [80], is a collection of methods to extract knowledge from opaque or “black box” machine learning methods like deep learning. XAI systems have been developed to meet this challenge, primarily motivated by image classification concerns like the erroneous classifications with the ruler in the image problem [79]. One example of an XAI method that opens the AI black box for interpretability is a saliency map [81]. A saliency map reveals information on the degree that each feature in the image explain and contribute to predictions [82]. Saliency maps applied in tandem with a deep convolution neural network can leverage the high degree of accurate predictions while retaining interpretable and explainable aspects of the underlying model. Another similar example of XAI used in tandem with a less explainable model occurs with random forests where one can compare the “variable importance” resulting from a comparison of the number of decision trees in which the variable appears, normalized by the associated node impurity decrease.

#### What are the available tools and how can they be used to model in nutrition?

XAI methods in nutrition are just beginning to advance [50, 83]. For example, XAI has been recently applied to automatic identification of food from images [84]. Food imaging and classification have been used in the Remote Food Photography Method [85] and in eating sensors [86, 87] and represent a novel objective method to estimate food intake in free-living humans.

## Data literacy: the AI user responsibility

An issue that is rarely addressed is the accountability of AI/ML consumers regarding data literacy. Because of our increasing reliance on AI/ML in nutrition, a certain level of data literacy and data standards needs to be embraced by all nutrition stakeholders. A critical component of data literacy is properly specifying a data-driven question and analyzing whether the question can be answered through descriptive analytics, diagnostic analytics, or predictive analytics. Further, as practitioners increase their data literacy they are better postured to combine the techniques given above. Indeed, many of the methods that fall under AI/ML are diverse and require specialized training. Even trained mathematical modelers cannot be experts in all possible methods and areas – just like any other discipline that interfaces with nutrition. Therefore, we advocate for more articles like the one presented here with checklists and summaries that help the nutrition research community address the right questions that will require models to be transparent, reproducible, and ethically applied.

## Conclusions

The quality of AI/ML modeling requires iterative and tailored processes to mitigate against potential ethical problems or to predict conclusions that are free of bias. Some of these feasibility checks may require a background in AI/ML training and including research team members with expertise will provide support for these analyses. Providing some basic best practice AI/ML modeling principles provides a path for researchers interested in using AI/ML models to understand and implement in nutrition applications.
